# Melanonychia Secondary to Long-Term Treatment with Hydroxycarbamide: An Essential Thrombocytosis Case

**DOI:** 10.1155/2015/653178

**Published:** 2015-08-02

**Authors:** Umit Yavuz Malkan, Gursel Gunes, Eylem Eliacik, Okan Yayar, Ibrahim Celalettin Haznedaroglu

**Affiliations:** Department of Hematology, School of Medicine, Hacettepe University, 0100 Ankara, Turkey

## Abstract

Hydroxycarbamide is used in the treatment of essential thrombocytosis and other myeloproliferative disorders. We report the case of a 63-year-old woman with essential thrombocytosis who had melanonychia after the long-term use of the hydroxycarbamide with a dose of 1000 mg/day. Two years after the initiation of the hydroxycarbamide, our patient had pain on her toes and melanonychia on her nails. Hydroxycarbamide treatment was discontinued because of pain and she was given anagrelide treatment. The pathogenesis of melanonychia secondary to long-term hydroxycarbamide treatment is not yet well understood. Some investigators suggested that genetic factors, induction of melanocytes, and some changes in nail matrix could be the reason of hydroxycarbamide related melanonychia. Our patient has suffered color changes in her nails as well as pain that made us doubtful for a beginning of ulceration besides melanonychia. Maybe early clinical reaction of discontinuation of the drug has prevented more severe side effect like ulceration in our patient. Also side effect of hydroxycarbamide has developed more slowly in our patient compared to other patients in the mentioned study. To conclude, long-term hydroxycarbamide treatment can cause mucocutaneous side effects and more studies should be done in future in order to reveal the underlying mechanism.

## 1. Introduction

Hydroxycarbamide is used in the treatment of essential thrombocytosis and other myeloproliferative disorders [1]. Melanonychia is one of the cutaneous abnormalities including nail changes that could develop with long-term treatment with hydroxycarbamide [2]. Herein, we aimed to present an essential thrombocytosis case with melanonychia that developed secondary to long-term hydroxycarbamide treatment.

## 2. Case Report

**Figure 1:**
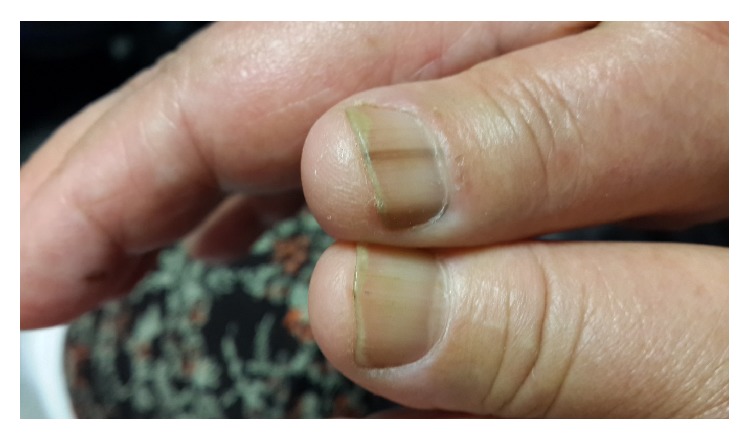
Melanonychia of the upper extremity nails.

**Figure 2:**
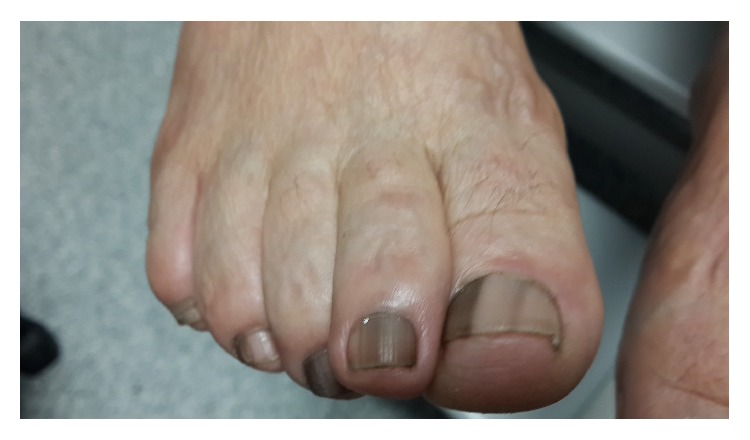
Melanonychia of the lower extremity nails.

63-year-old woman applied to hospital in July 2012 with bruises on chest without trauma. In her anamnesis, it was learned that she also had large bruises after trauma before the admission. In her physical examination spleen was palpable under arcus costa. Her laboratory tests results were hemoglobin 13.6 gr/dL, white blood cell 8.2 × 10^3^/*μ*L, and platelet 600 × 10^3^/*μ*L. There was no cause for reactive thrombocytosis and serum iron levels were normal. Hematocrit level was 37%. In detailed tests Philadelphia chromosome was absent and JAK2 mutation was positive. She was diagnosed as having essential thrombocytosis and she was given hydroxycarbamide treatment with a dose of 2 × 500 mg/day. There were no complications and she was on periodic follow-up by our clinic. However in July 2014 she started to feel pain in her toes. Clinical examination revealed melanonychia on her upper and lower extremity nails (Figures 1 and 2). Hydroxycarbamide treatment was discontinued because of pain and she was given Anagrelide treatment. Anagrelide was well tolerated and our patient is still on clinical follow-up with this treatment without complication.

## 3. Discussion

Melanocytes are located on the matrix of the nail and they are latent until receiving an activating signal. After activation melanin starts to increase and then melanocytes become visible in the nail plate. Melanonychia is associated with race and age [3, 4]. The treatment of high risk essential thrombocytosis cases can be managed by hydroxycarbamide, interferon-*α*, and Anagrelide [5–8]. The pathogenesis of melanonychia secondary to long-term hydroxycarbamide treatment is not yet well understood. The mechanism of the involvement of nail is not clear [9]. It seems that hydroxycarbamide has an activating effect on melanocytes [10]. In the literature, some investigators suggested that genetic factors, induction of melanocytes, and some changes in nail matrix could be the reason of hydroxycarbamide related melanonychia [9, 11]. Some authors suggest that photosensitivity and UV radiation could be some of the causes of melanonychia secondary to hydroxycarbamide usage [12]. Also some authors suggest that women have a tendency to melanonychia secondary to hydroxycarbamide usage [13]. There are two reports of case series with 9 and 7 patients, respectively, in the literature presenting melanonychia in essential thrombocytosis patients who had been treated with hydroxycarbamide [12, 14]. In these studies side effect of melanonychia was not found to be related with the treatment dosage or period of the hydroxycarbamide treatment. Both studies report melanonychia could be seen in the nails of both hand and foot. One of these reports has stated that although melanonychia could be seen in upper and lower extremity nails, nails of hand seemed to be affected more than foot [12]. Different from this study, in our patient melanonychia was more apparent in the nails of foot than hand. In a study that was conducted with 993 patients of myeloproliferative neoplasms (half of them were essential thrombocytosis) treated with hydroxycarbamide, mucocutaneous side effects were reported in 8.3% of the patients [15]. These mucocutaneous side effects include skin ulcers, oral ulcers, and skin infiltration. In this study, median time for occurrence of early toxicities was found as 2.1 months after initiation and median time for occurrence of late toxicities was found as 38 months after initiation of hydroxycarbamide. It was also reported in this study that nearly 50% of the patients discontinued hydroxycarbamide permanently because of side effects. Our patient has suffered color changes in her nails as well as pain that made us doubtful of a beginning of ulceration besides melanonychia. Maybe early clinical reaction of discontinuation of the drug has prevented more severe side effect like ulceration in our patient. Also side effect of hydroxycarbamide has developed more slowly in our patient compared to other patients in the mentioned study. To conclude, long-term hydroxycarbamide treatment can cause mucocutaneous side effects and more studies should be done in future in order to reveal the underlying mechanism.

## References

[1] Boyd A. S., Neldner K. H. (1991). Hydroxyurea therapy. *Journal of the American Academy of Dermatology*.

[2] Vomvouras S., Pakula A. S., Shaw J. M. (1991). Multiple pigmented nail bands during hydroxyurea therapy: an uncommon finding. *Journal of the American Academy of Dermatology*.

[3] Duhard E., Clavet C., Mariotte N., Tichet J., Vaillant L. (1995). Prevalence of longitudinal melanonychia in white subjects. *Annales de Dermatologie et de Venereologie*.

[4] Baran R., Kechijian P. (1989). Longitudinal melanonychia (melanonychia striata): diagnosis and management. *Journal of the American Academy of Dermatology*.

[5] Beer P. A., Green A. R. (2009). Pathogenesis and management of essential thrombocythemia. *Hematology/The Education Program of the American Society of Hematology. American Society of Hematology. Education Program*.

[6] Cervantes F. (2011). Management of essential thrombocythemia. *ASH Hematology, the Education Program*.

[7] Birgegård G. (2013). Pharmacological management of essential thrombocythemia. *Expert Opinion on Pharmacotherapy*.

[8] Tefferi A., Barbui T. (2013). Personalized management of essential thrombocythemia—application of recent evidence to clinical practice. *Leukemia*.

[9] Teo R. Y. L., Tan E. (2006). A case of hydroxyurea-induced transverse melanonychia. *International Journal of Dermatology*.

[10] Kelsey P. R. (1992). Multiple longitudinal pigmented nail bands during hydroxyurea therapy. *Clinical and Laboratory Haematology*.

[11] Issaivanan M., Mitu P. S., Chakrabarti M., Khairkar P. (2004). Cutaneous manifestations of hydroxyurea therapy in childhood: case report and review. *Pediatric Dermatology*.

[12] Murray N. P., Tapia P., Porcell J., Echavarria M., Suazo H. (2013). Acquired melanonychia in chilean patients with essential thrombocythemia treated with hydroxyurea: a report of 7 clinical cases and review of the literature. *ISRN Dermatology*.

[13] Neynaber S., Wolff H., Plewig G., Wienecke R. (2004). Longitudinal melanonychia induced by hydroxyurea therapy. *Journal der Deutschen Dermatologischen Gesellschaft*.

[14] Aste N., Fumo G., Contu F., Aste N., Biggio P. (2002). Nail pigmentation caused by hydroxyurea: report of 9 cases. *Journal of the American Academy of Dermatology*.

[15] Latagliata R., Spadea A., Cedrone M. (2012). Symptomatic mucocutaneous toxicity of hydroxyurea in Philadelphia chromosome-negative myeloproliferative neoplasms the mister hyde face of a safe drug. *Cancer*.

